# PCYT1B-Targeting miRNAs as Potential Biomarkers for Placental Diseases

**DOI:** 10.3390/ijms27094039

**Published:** 2026-04-30

**Authors:** Ha Eun Shin, Jin Seok, Jae Yeon Kim, Dong-Hyun Cha, Joong Sik Shin, Gi Jin Kim

**Affiliations:** 1 Department of Biomedical Science, CHA University, Seongnam-si 13488, Republic of Korea; ttorumaru@gmail.com; 2 Research Institute of Placental Science, CHA University, Seongnam-si 13488, Republic of Korea; jjin8977@gmail.com; 3 PLABiologics Co., Ltd., Seongnam-si 13522, Republic of Korea; 4 Division of Life Sciences, Department of Life Science, Graduate School, CHA University, Seongnam-si 13488, Republic of Korea; janejykim92@gmail.com; 5 Department of Obstetrics and Gynecology, CHA General Hospital, CHA University, Seoul 06135, Republic of Korea; chadh001@chamc.co.kr (D.-H.C.); shinjs@cha.ac.kr (J.S.S.)

**Keywords:** miRNA, PCYT1B, pre-eclampsia, preterm labor, invasion

## Abstract

Obstetrical diseases are complications associated with pregnancy or childbirth that can cause maternal sequelae and fetal complications. Among them, preeclampsia (PE) and preterm labor (PTL) are major causes of premature birth and are associated with an increased risk of cerebral palsy, developmental delay, and hearing impairment in infants. However, reliable diagnostic markers and therapeutic strategies for obstetrical diseases remain limited. The aim of this study was to investigate genes associated with obstetrical diseases and to evaluate the correlation between phosphocholine cytidylyltransferase 1 beta (PCYT1B) and miRNAs targeting PCYT1B for diagnostic analysis in PE and PTL. Using miRNA array analysis and luciferase assays, we identified PCYT1B, a key enzyme involved in phosphocholine metabolism in reproductive tissues, together with several candidate miRNAs targeting PCYT1B, including miR-3065-3p, miR-4660, miR-6752-5p, miR-6842-5p and miR-7110-5p. qRT-PCR analysis revealed a significant correlation between *PCYT1B* and these miRNAs in placental tissues from patients with PE and PTL (*p* < 0.05). Immunofluorescence staining further demonstrated that PCYT1B was localized in the syncytiotrophoblast layer of placental tissues, and its protein expression was consistent with mRNA expression levels. To investigate the functional role of these miRNAs, trophoblast cells were treated with miRNA mimics and inhibitors. These treatments significantly altered trophoblast invasion capacity and regulated the expression of migration-related genes, including *RhoA*, *Rac1* and *ROCK*. Collectively, our findings suggest that miRNAs targeting PCYT1B may regulate trophoblast function and may play a key role in placental development and obstetrical diseases. These results indicate that PCYT1B and its regulatory miRNAs could serve as potential biomarkers for PE and PTL and may provide insights into the development of miRNA-based diagnostic strategies.

## 1. Introduction

During pregnancy, proper placental development is essential for maintaining a healthy gestational environment. The placenta functions as a critical interface between the maternal and fetal systems, enabling the exchange of gases, nutrients, and metabolic waste. In addition, it plays an essential role in regulating maternal–fetal immune interactions necessary for maintaining pregnancy [1,2]. However, disruption of placental function can lead to several pregnancy-related complications, including miscarriage, preterm labor (PTL), and preeclampsia (PE).

PTL is defined as the delivery before 37 weeks of gestation and is primarily caused by premature uterine contractions. It is a major contributor to perinatal mortality and long-term morbidity, including chronic respiratory disorders, neurodevelopmental impairments, and cognitive dysfunction. Despite its clinical significance, the molecular mechanisms underlying PTL remain incompletely understood, and reliable predictive biomarkers for early identification are still limited [3,4]. PTL is considered a multifactorial syndrome; although some cases are associated with intra-amniotic infection, increasing evidence suggests that placental insufficiency contributes to a substantial proportion of PTL cases [5,6]. Similarly, PE is a pregnancy-specific disorder characterized by hypertension and multi-organ dysfunction, which significantly increases the risk of maternal and fetal morbidity and mortality. The etiology of PE has been linked to multiple factors, including genetic predisposition, immune dysregulation, and maternal comorbidities. These factors can induce abnormal placentation characterized by defective trophoblast differentiation and insufficient trophoblast invasion. Consequently, impaired spiral artery remodeling results in inadequate uteroplacental circulation and maternal hypertension [7]. Despite the clinical importance of PTL and PE, reliable diagnostic biomarkers and effective therapeutic strategies remain limited.

Phosphocholine cytidylyltransferase 1 beta (PCYT1B) is highly expressed in the placenta and testis and plays an important role in phosphocholine metabolism and phosphatidylcholine biosynthesis [8]. Phosphatidylcholine is a major component of cellular membranes and is essential for maintaining membrane permeability, receptor activity, enzyme function, and structural stability through protein-lipid interactions [9,10]. Previous studies have reported that phospholipids, particularly phosphocholine, are highly abundant in reproductive tissues such as the myometrium, decidua, placenta, and fetal membranes during pregnancy. Furthermore, the transplacental transport of nutrients from mother to fetus occurs across the basal plasma membrane of syncytiotrophoblast cells [11,12]. In addition, phosphocholine-related pathways have been implicated in inflammatory responses mediated by C-reactive protein (CRP) and endothelial cell migration and angiogenesis [13,14,15]. However, the expression patterns and functional roles of PCYT1B in placental tissues under obstetrical disease conditions remain unexplored.

MicroRNAs(miRNAs) are small non-coding RNA molecules that regulate gene expression at the post-transcriptional level and play key roles in various physiological and pathological processes. Circulating miRNAs in plasma and serum are stable and have attracted attention as potential biomarkers for numerous diseases [16,17,18]. Several circulating miRNAs have been reported to be placenta-specific and are involved in regulating pregnancy-related processes, including inflammatory responses and hypoxic adaptation [19,20]. In addition, previous studies have demonstrated that specific miRNAs regulate angiogenesis and exhibit differential expression patterns in PTL and PE [21,22]. Because circulating miRNAs can be readily detected in body fluids, they represent promising candidates for non-invasive diagnostic biomarkers. However, current studies on miRNA-based biomarkers remain limited by heterogeneous origin of circulating miRNAs and the incomplete understanding of their molecular mechanisms.

In the present study, we investigated miRNA profiles targeting PCYT1B in placental tissues and maternal plasma from patients with PTL and PE. Furthermore, we evaluated the expression levels of PCYT1B and its associated miRNAs to determine their potential as biomarkers for early diagnosis and prognosis of obstetrical diseases.

## 2. Results

### 2.1. Profiling and Identification of miRNAs Targeting PCYT1B in Obstetrical Diseases

**Figure 1:**
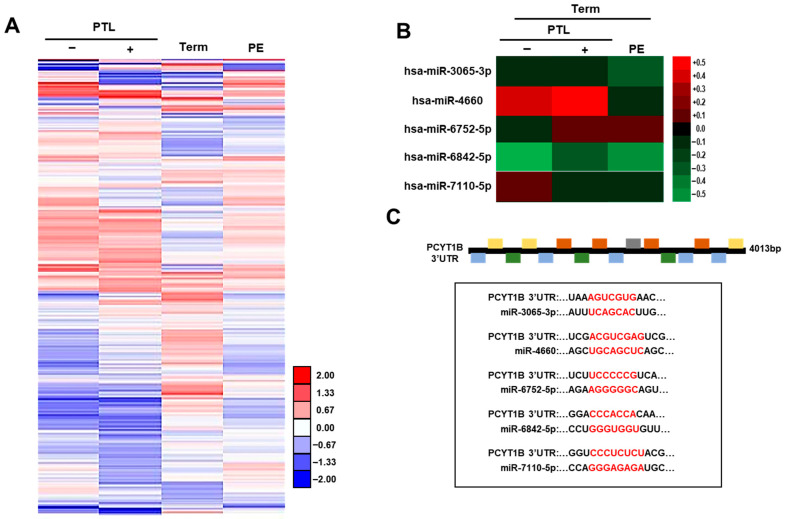
Heatmap analysis and predicted interactions between PCYT1B and selected miRNAs in obstetrical diseases. (**A**) Heatmap of cDNA microarray data showing differentially expressed genes associated with preeclampsia (PE) and preterm labor (PTL) in human placental tissues from each group. (**B**) Heatmap of miRNA microarray data showing the expression profiles of selected candidate miRNAs predicted to target PCYT1B in placental tissues from each group. (**C**) Predicted binding sites of the selected miRNAs within the 3′ untranslated region (3′UTR) of PCYT1B, including miR-3065-3p, miR-4660, miR-6752-5p, miR-6842-5p and miR-7110-5p.

To identify genes and miRNAs associated with obstetrical diseases, including preterm labor (PTL) and preeclampsia (PE), we performed cDNA and miRNA expression profiling using human placental tissues obtained from patients. Among the differentially expressed genes identified by cDNA microarray analysis, phosphocholine cytidylyltransferase 1 beta (PCYT1B), a key enzyme involved in phosphatidylcholine biosynthesis, was selected as a candidate gene based on its altered expression pattern (Figure 1A).

To identify miRNAs targeting PCYT1B, we integrated data from public miRNA databases (miRbase; www.mirbase.org) with our miRNA microarray results. Based on this analysis, five candidate miRNAs—miR-3065-3p, miR-4660, miR-6752, miR-6842-5p, and miR-7110-5p—were selected as potential upstream regulators of PCYT1B. Heatmap analysis revealed a significant correlation between PCYT1B expression and the selected miRNAs in placental tissues (Figure 1B).

To further assess their potential interaction, we analyzed the predicted binding sites within the 3′ untranslated region (3′UTR) of PCYT1B mRNA. As shown in Figure 1C, all selected miRNAs were predicted to bind to specific regions of the PCYT1B 3′UTR. These findings suggest that the identified miRNAs may regulate PCYT1B expression and may be involved in the pathophysiology of obstetrical diseases such as preterm labor and preeclampsia.

### 2.2. Interactions Between Selected miRNAs and PCYT1B in Trophoblast Cells

**Figure 2:**
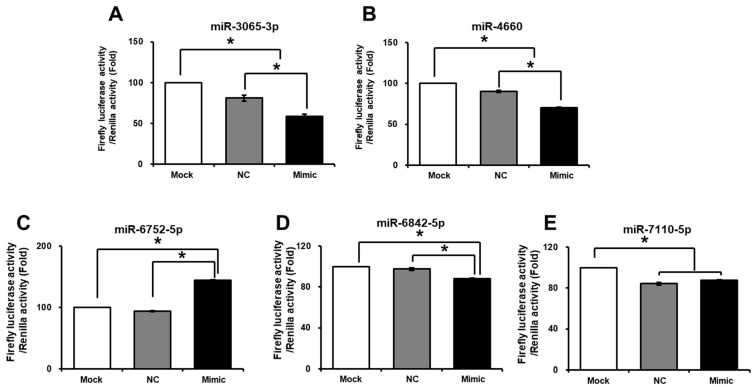
Validation of interactions between selected miRNAs and PCYT1B using a dual-luciferase reporter assay. (**A**–**E**) Relative luciferase activity following co-transfection of the PCYT1B 3′UTR reporter construct with miRNA mimics for (**A**) miR-3065-3p, (**B**) miR-4660, (**C**) miR-6752-5p, (**D**) miR-6842-5p, and (**E**) miR-7110-5p. Data are presented as mean ± SEM. Statistical significance was determined using Student’s *t*-test. Mock, mock-transfected control; NC, negative control miRNA; mimic, cell transfected with each miRNA mimic. * *p* < 0.05 was considered statistically significant.

Based on the previous profiling results, we predicted a regulatory relationship between PCYT1B and the selected miRNAs. To determine whether PCYT1B is a direct target of these miRNAs, a dual-luciferase reporter assay was performed using constructs containing the 3′ untranslated region (3′UTR) of PCYT1B. HTR-8/SVneo cells were co-transfected with the PCYT1B 3′UTR reporter vector and each miRNA mimic. As shown in Figure 2A,B,D, luciferase activity was significantly decreased in cells transfected with miR-3065-3p, miR-4660, and miR6842-5p mimics compared with the mock and negative control groups (*p* < 0.05; Figure 2C). For miR-7110-5p, luciferase activity was significantly reduced compared with the mock group, although no significant difference was observed between the negative control and mimic-treated groups (*p* < 0.05; Figure 2E). These results suggest that the selected miRNAs directly interact with the 3′UTR of PCYT1B and may regulate its expression.

### 2.3. Validation of PCYT1B and Selected miRNAs in Preterm Labor

**Figure 3:**
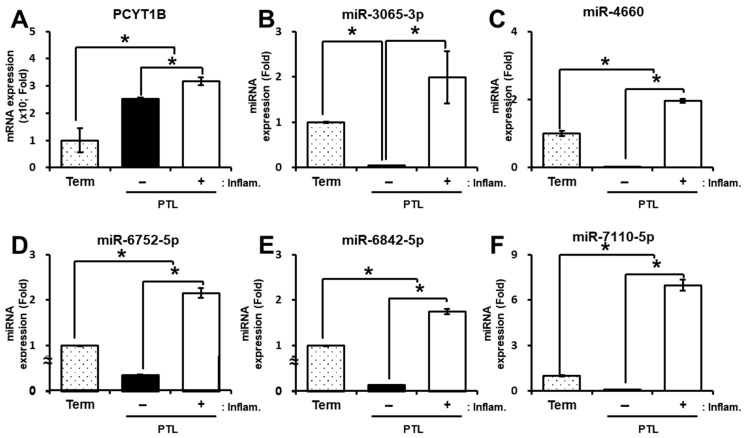
Expression of PCYT1B and selected miRNAs in maternal plasma from patients with preterm labor (PTL). (**A**) Relative mRNA expression of *PCYT1B* in maternal plasma. (**B**–**F**) Relative expression levels of selected miRNAs in maternal plasma: (**B**) miR-3065-3p, (**C**) miR-4660, (**D**) miR-6752-5p, (**E**) miR-6842-5p, and (**F**) miR-7110-5p. Data are presented as mean ± SEM. Statistical significance was determined using Student’s *t*-test. * *p* < 0.05 was considered statistically significant.

To investigate the association between PCYT1B and the selected miRNAs in preterm labor (PTL), we analyzed their expression levels in plasma and placental tissues obtained from patients with PTL. In plasma samples, *PCYT1B* mRNA expression was significantly increased in PTL cases with or without inflammation compared with the term control group (*p* < 0.05; Figure 3A). We next examined the expression levels of the selected miRNAs in plasma samples from PTL patients. The selected miRNAs showed reduced expression in PTL without inflammation compared with the term group. In contrast, PTL cases with inflammation exhibited increased miRNA expression compared with both the PTL without inflammation and term group (*p* < 0.05, Figure 3B–F).

**Figure 4:**
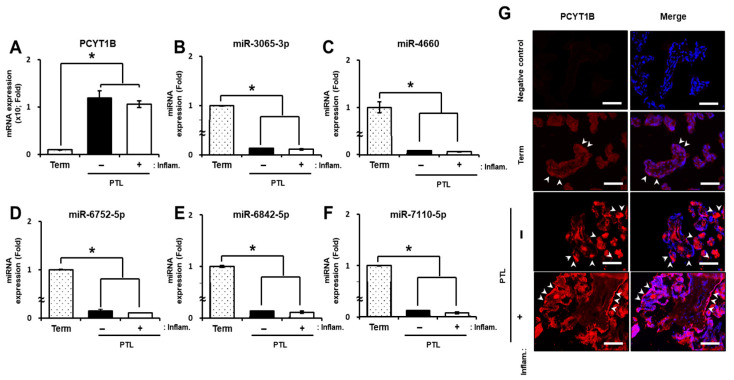
Expression of PCYT1B and selected miRNAs in placental tissues from preterm labor (PTL) patients. (**A**) Relative mRNA expression of *PCYT1B*. (**B**–**F**) Relative expression levels of selected miRNAs: (**B**) miR-3065-3p, (**C**) miR-4660, (**D**) miR-6752-5p, (**E**) miR-6842-5p and (**F**) miR-7110-5p in placental tissue. (**G**) Representative immunofluorescence images showing PCYT1B expression in placental tissues. Nuclei were counterstained with DAPI (blue). Merge indicates the overlays of PCYT1B staining and DAPI. White arrows indicate PCYT1B-positive trophoblast cells. Original magnification, ×200. Scale bar = 100 µm. Data are presented as mean ± SEM. Statistical significance was determined using Student’s *t*-test. * *p* < 0.05 was considered statistically significant.

We further evaluated PCYT1B and miRNA expression in placental tissues from PTL patients. As shown in Figure 3A, *PCYT1B* mRNA expression was significantly increased in PTL placental tissues regardless of inflammatory status compared with term placentas (*p* < 0.05; Figure 4A). In contrast, the selected miRNAs showed significantly decreased expression in PTL placental tissues compared with term controls (*p* < 0.05; Figure 4B–F). These findings indicate that PCYT1B and the selected miRNAs exhibit a stronger inverse expression pattern in placental tissues than in plasma samples from PTL patients. 

To further examine the expression and localization of PCYT1B in placental tissues, immunofluorescence staining was performed. As shown in Figure 4G, PCYT1B was localized in the syncytiotrophoblast layer of the placenta. Consistent with the mRNA expression results, PCYT1B protein expression was significantly increased in PTL placentas compared with term controls (*p* < 0.05; Figure 4G). Together, these results suggest that PCYT1B and its associated miRNAs are differentially expressed in PTL placental tissues and may be involved in the pathophysiology of preterm labor.

### 2.4. Validation of PCYT1B and Selected miRNAs in Preeclampsia

**Figure 5:**
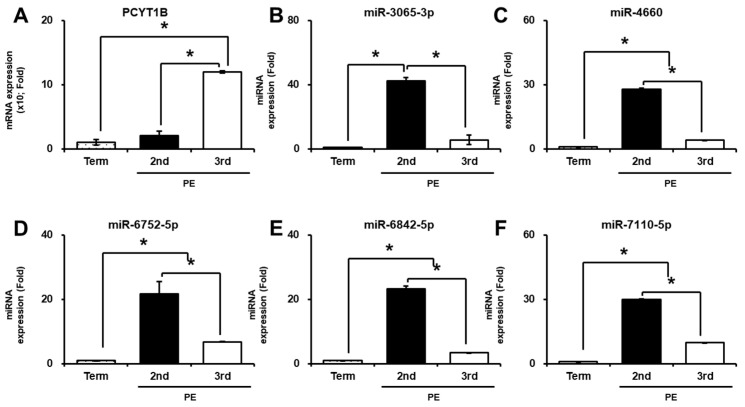
Expression of PCYT1B and selected miRNAs in maternal plasma from patients with preeclampsia (PE). (**A**) Relative mRNA expression of *PCYT1B* in maternal plasma. (**B**–**F**) Relative expression levels of selected miRNAs in maternal plasma: (**B**) miR-3065-3p, (**C**) miR-4660, (**D**) miR-6752-5p, (**E**) miR-6842-5p, and (**F**) miR-7110-5p. Data are presented as mean ± SEM. Statistical significance was determined using Student’s *t*-test. * *p* < 0.05 was considered statistically significant.

To investigate the association between PCYT1B and the selected miRNAs in preeclampsia (PE), their expression levels were analyzed in plasma and placental tissues from PE patients. In plasma samples *PCYT1B* mRNA expression was significantly increased in both second- and third-trimester PE compared with term controls (*p* < 0.05; Figure 5A). We next examined the expression of the selected miRNAs in plasma samples. The selected miRNAs—including miR-3065-3p, miR-4660, miR-6752-5p, miR-6842-5p, and miR-7110-5p—showed increased expression in both second- and third-trimester PE compared with term controls. Notably, miRNA expression levels were significantly higher in second-trimester PE than in third-trimester PE (*p* < 0.05, Figure 5B–F).

**Figure 6:**
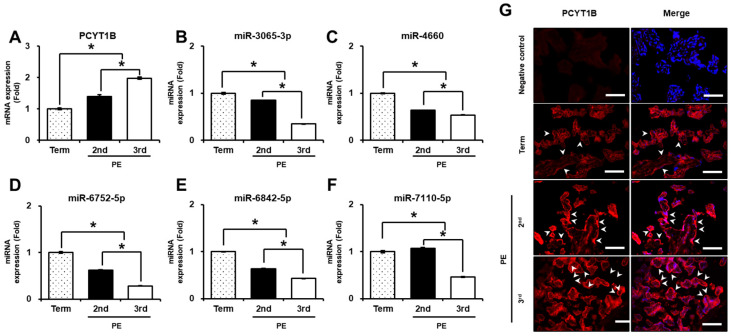
Expression of PCYT1B and selected miRNAs in placental tissues from patients with pre-eclampsia (PE). (**A**) Relative mRNA expression of *PCYT1B*. (**B**–**F**) Relative expression levels of selected miRNAs: (**B**) miR-3065-3p, (**C**) miR-4660, (**D**) miR-6752-5p, (**E**) miR-6842-5p and (**F**) miR-7110-5p in placental tissues. (**G**) Representative immunofluorescence images showing PCYT1B expression in placental tissues. Nuclei were counterstained with DAPI (blue). Merge indicates the overlays of PCYT1B staining and DAPI. White arrows indicate PCYT1B-positive trophoblast cells. Original magnification, ×200. Scale bar = 100 µm. Data are presented as mean ± SEM. Statistical significance was determined using Student’s *t*-test. * *p* < 0.05 was considered statistically significant.

We further evaluated PCYT1B and miRNA expression in placental tissues from PE patients. As shown in Figure 6A, *PCYT1B mRNA expression* was significantly increased in placental tissues from both second- and third-trimester PE compared with term placentas (*p* < 0.05; Figure 6A). In contrast, the expression levels of miR-3065-3p, miR-4660, miR-6752-5p, and miR-6842-5p were significantly decreased in PE placental tissues compared with term controls (*p* < 0.05; Figure 6B–E).

Within the PE groups, miRNA expression levels were significantly lower in third-trimester PE compared with second-trimester PE. For miR-7110-5p, expression was slightly increased in second-trimester PE compared with term controls, whereas a significant decrease was observed in third-trimester PE compared with both term and second-trimester PE samples (*p* < 0.05; Figure 6F). Overall, these findings indicate that PCYT1B and the selected miRNAs exhibit distinct expression patterns in plasma and placental tissues in PE, with a clearer inverse relationship observed in placental tissues.

To further examine the expression and localization of PCYT1B, immunofluorescence staining was performed. As shown in Figure 6G, PCYT1B was localized in the syncytiotrophoblast layer of placental tissues. In addition, PCYT1B protein expression was significantly increased in both second- and third-trimester PE compared with term controls (Figure 6G). These results were consistent with the mRNA expression patterns observed in placental tissues from PE patients. Together, these findings suggest that PCYT1B and the selected miRNAs exhibit correlated expression patterns in placental tissues in preeclampsia.

### 2.5. Effect of Selected miRNAs and PCYT1B on Trophoblast Invasion

Trophoblast invasion plays a critical role in placental development during early implantation. Impaired trophoblast invasion has been associated with pregnancy-related disorders such as preterm labor (PTL) and preeclampsia (PE) [23,24]. Therefore, we investigated whether PCYT1B and the selected miRNAs regulate the invasion ability of trophoblast cells.

**Figure 7:**
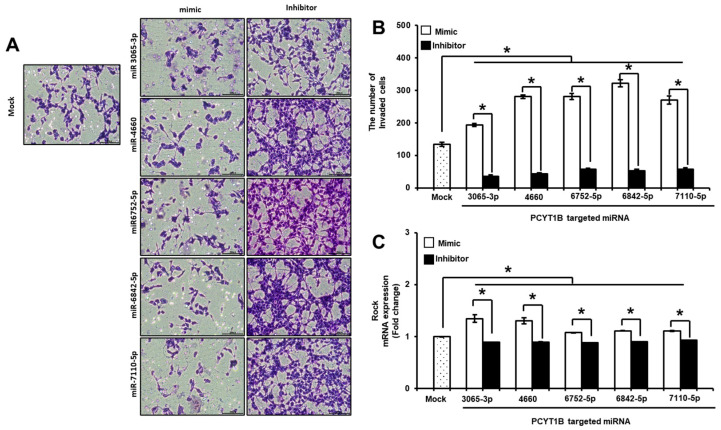
Effect of PCYT1B-targeting miRNAs on trophoblast cell invasion and migration-related gene expression. HTR-8/SVneo trophoblast cells were transfected with miRNA mimics or inhibitors targeting PCYT1B. (**A**) Representative images showing invaded trophoblast cells in the Trans-well invasion assay following transfection with miRNA mimics or inhibitors. (**B**) Quantification of invaded cells using ImageJ software (National Institutes of Health, Bethesda, MD, USA; version1.53). (**C**) Relative mRNA expression of *ROCK* analyzed by qRT-PCR after treatment with miRNA mimics or inhibitors. Data are presented as mean ± SEM. Statistical significance was determined using Student’s *t*-test. * *p* < 0.05 was considered statistically significant.

Based on the previous results, HTR-8/SVneo trophoblast cells were treated with miRNA mimics or inhibitors to evaluate their functional effects on PCYT1B expression and trophoblast invasion. Following miRNA mimic transfection, the expression levels of the selected miRNAs were significantly increased, whereas PCYT1B expression was decreased compared with the mock control. In contrast, miRNA inhibitor treatment resulted in decreased miRNA expression and increased PCYT1B expression (*p* < 0.05; Figure S1). We next examined the invasion ability of trophoblast cells. As shown in Figure 7A, trophoblast cells transfected with miRNA mimics exhibited increased invasion activity compared with mock controls, whereas miRNA inhibitor treatment significantly reduced invasion activity (Figure 7A). Quantitative analysis further confirmed that the number of invaded cells significantly increased in the mimic-treated group and decreased in the inhibitor-treated group (*p* < 0.05; Figure 7B). To further investigate the molecular mechanisms underlying these changes, we analyzed the expression of migration- and invasion-related genes, including *RhoA*, *Rac1*, and *ROCK*. The mRNA expression levels of RhoA, Rac1, and ROCK were significantly increased following miRNA mimic treatment compared with mock controls, whereas inhibitor treatment resulted in decreased expression of these genes (*p* < 0.05; Figures S1A,B and S2 and Figure 7C). Together, these findings suggest that PCYT1B-targeting miRNAs regulate trophoblast invasion and may contribute to placental development.

## 3. Discussion

Obstetrical diseases including preterm labor (PTL) and preeclampsia (PE), remain major causes of maternal and neonatal morbidity and mortality during pregnancy. Despite their clinical importance, reliable early diagnostic biomarkers and effective targeted therapies remain limited. Therefore, considerable efforts have focused on identifying circulating biomarkers, microRNAs (miRNAs), and exosomes in maternal blood. Several studies have reported differential miRNA expression in placental tissues and maternal circulation under pathological pregnancy conditions [25,26,27,28,29].

In the present study, integrated gene and miRNA array analyses identified phosphocholine cytidylyltransferase 1 beta (PCYT1B) as a candidate molecule associated with obstetrical diseases. PCYT1B expression was significantly increased in both placental tissues and maternal circulation from patients with PTL and PE, suggesting its involvement in placental dysfunction and its potential value as a biomarker candidate.

PCYT1B is a key regulatory enzyme in the Kennedy pathway and plays an essential role in phosphatidylcholine biosynthesis and lipid homeostasis [30]. Previous studies have shown that PCYT1B is involved in hepatic lipid metabolism through regulation of very low-density lipoprotein secretion. In reproductive tissues, disruption of PCYT1B has been associated with gonadal dysfunction, altered reproductive hormone levels, and impaired fertility potential [31]. In addition, PCYT1B-related pathways involving neuropathy target esterase (NTE) have been linked to endothelial dysfunction and CRP-associated preeclampsia [13,14,15]. 

These findings may be particularly relevant in placental biology. During pregnancy, phospholipids—especially phosphocholine—are highly enriched in the myometrium, decidua, placenta, and fetal membranes [12]. Maternal–fetal nutrient transport occurs through the basal plasma membrane of syncytiotrophoblast cells [11]. Membrane lipid composition strongly influences membrane integrity, receptor signaling, permeability, and structural stability [9,10,32]. Therefore, dysregulation of PCYT1B may alter placental membrane homeostasis and trophoblast function.

A possible mechanistic link between PCYT1B, the identified miRNAs, and obstetrical diseases may involve altered phosphatidylcholine metabolism and impaired trophoblast invasion. Because PCYT1B regulates phosphatidylcholine synthesis, its abnormal expression may affect membrane remodeling and cytoskeletal dynamics. Changes in membrane lipid composition may subsequently influence migration-related signaling pathways such as RhoA and ROCK, which are central regulators of cell motility and actin organization [33]. The identified miRNAs may regulate PCYT1B expression at the post-transcriptional level, thereby modulating trophoblast migration and contributing to abnormal placentation in PTL and PE.

Previous studies have linked some of these miRNAs to cancer progression [34], endothelial proliferation and migration [35], whereas others have been investigated as circulating biomarkers in cerebrovascular or inflammatory disorders [36,37,38]. However, their roles in placental pathology have not been previously clarified. Luciferase reporter assays confirmed direct interactions between these miRNAs and the 3′UTR of PCYT1B. Furthermore, inverse expression patterns between the selected miRNAs and PCYT1B were observed in clinical placental samples, suggesting loss of post-transcriptional regulation under pathological conditions. These findings support the presence of a miRNA-PCYT1B regulatory axis associated with placental dysfunction.

PCYT1B and its associated miRNAs may serve as potential biomarkers for PTL and PE for several reasons. First, PCYT1B was consistently elevated in placental tissues and maternal circulation from patients with obstetrical diseases. Second, the identified miRNAs showed disease-associated differential expression patterns and direct regulatory interactions with PCYT1B. Third, circulating miRNAs can be detected through minimally invasive blood-based approaches, which may enable early screening and disease monitoring during pregnancy. Therefore, combined assessment of PCYT1B and its targeting miRNAs may support the development of miRNA-based diagnostic strategies and improve the clinical utility of biomarker screening for PTL and PE.

Several limitations should be considered. First, although this study identified the PCYT1B-miRNA axis as a potential regulator of trophoblast invasion and placental dysfunction, the precise downstream molecular mechanisms remain unclear. Second, the small cohort size may limit the generalizability of the findings. In addition, larger validation cohorts will be required to perform ROC curve analyses and to determine the diagnostic sensitivity and specificity of PCYT1B-associated miRNAs in PTL and PE. Third, PTL and PE are heterogeneous disorders, and larger multicenter studies together with in vivo models are needed to determine whether this regulatory axis represents a causal mechanism or a secondary consequence of placental pathology.

Taken together, this study demonstrated a potential regulatory relationship between PCYT1B and its associated miRNAs through integrated transcriptomic profiling, clinical validation, luciferase assays, and functional invasion analyses. Our findings provide new insights into placental dysfunction and suggest that PCYT1B and its associated miRNAs may represent promising candidates for biomarker development and therapeutic targets in pregnancy-related disorders.

## 4. Materials and Methods

### 4.1. Human Placenta and Blood Sample Collection from Pre-Eclampsia and Preterm Labor Patients

For the analysis of PE, sample collection and use were approved by the Institutional Review Board of CHA General Hospital, Seoul, Republic of Korea (IRB No. 006-12). The clinical characteristics of the PE and control groups are summarized in Table 1. The mean age of normal pregnant women (NPE, n = 10) was 34 ± 2 years with an average gestational age of 38 ± 2.5 weeks. Their mean systolic and diastolic blood pressures were 115 ± 3 mmHg and 72 ± 2 mmHg, respectively. No proteinuria was detected in the normal pregnancy group. The PE group included 15 women diagnosed with preeclampsia during the third trimester. The mean age of PE patients was 30.2 ± 1.1 years, and the mean gestational age was 37 ± 3.4 weeks. Their average systolic and diastolic blood pressures were 134 ± 7 mmHg and 91 ± 2 mmHg, respectively. Proteinuria levels detected by dipstick analysis were greater than two positive readings. 

For the analysis of PTL, the collection of human placental tissues and their use for research purposes were approved by the Institutional Review Board of Seoul National University College of Medicine, Seoul, Republic of Korea (IRB No. H-1105-045-361). Written informed consent was obtained from all participants prior to sample collection. Normal term placentas (n = 5) were obtained from women without medical or surgical complications who delivered at term (≥37 gestational weeks). PTL placentas were collected from women who delivered preterm (≤35 gestational weeks), including cases with inflammation (n = 4) and without inflammation (n = 6). The characteristics of the PTL groups are summarized in Table 2.

Placental tissues and maternal blood samples were additionally classified into the following groups: (1) normotensive women who delivered at term without labor by elective cesarean section (n = 15); (2) women with term preeclampsia who delivered without labor by cesarean section (n = 15); and (3) women with preterm preeclampsia who delivered without labor by cesarean section (n = 11). All placental tissues were obtained from the central region of the placenta, immediately snap-frozen in liquid nitrogen, and stored at −80 °C until analysis.

### 4.2. HTR-8/SVneo Cell Culture and miRNA Mimic/Inhibitor Transfection

The HTR-8/SVneo trophoblast cell line was kindly provided by Dr. Graham (Queen’s University, Kingston, ON, Canada). Cells were cultured in Roswell Park Memorial Institute medium (RPMI-1640; Hyclone, GE Healthcare Life Sciences, Logan, UT, USA) supplemented with 5% fetal bovine serum (FBS; Gibco, Rockville, MD, USA) and 1% penicillin-streptomycin (Pen-Strep; Gibco, Rockville, MD, USA). Cells were maintained at 37 °C in a humidified incubator containing 5% CO_2_. 

To evaluate whether selected miRNAs regulate PCYT1B expression, HTR-8/SVneo cells were seeded in 24-well plates and transfected with miRNA mimic or inhibitor of 50 nM using Lipofectamine 2000 (Invitrogen, Carlsbad, CA, USA) according to the manufacturer’s protocol. Cells were incubated for 24 h prior to downstream analyses.

### 4.3. RNA Isolation and Quantitative Real-Time Polymerase Chain Reaction

Total RNA was extracted from human placental tissues using Trizol reagent (Ambion, Boston, MA, USA) according to the manufacturer’s instructions. RNA concentration and purity were measured using Nanodrop 2000 (Thermo Fisher Scientific, Waltham, MA, USA). For cDNA synthesis, 500 ng of total RNA was reverse transcribed using Superscript III RNase H reverse transcriptase (Invitrogen, Carlsbad, CA, USA) following the manufacturer’s protocol. Briefly, first-strand cDNA synthesis was performed using oligo dT primers (Invitrogen) and dNTP mix (Thermo Fisher Scientific). The reaction mixture included DTT, RNaseOut, Superscript III enzyme, and 5× first-strand buffer (Thermo Fisher Scientific).

Quantitative real-time PCR was performed under the following conditions: an initial denaturation at 95 °C for 10 min, followed by 40 to 45 cycles of 95 °C for 15 s and 60 °C for 30 s. All reactions were performed in duplicate or triplicate. Relative mRNA expression levels were calculated using the comparative CT (2^−∆∆Ct^) method. Primer sequences used in this study are listed in Table 3.

### 4.4. miRNA Extraction and Reverse Transcription-PCR

Total RNA was extracted from 200 µL of human serum using the miRNeasy Serum/Plasma kit (Qiagen, Hilden, Germany) according to the manufacturer’s instructions. Briefly, 200 µL of serum was mixed with 1 mL of QIAzol lysis reagent and incubated at room temperature for 5 min. Subsequently, 3.5 µL of miRNeasy Serum/Plasma Spike-In Control (1.6 × 10^8^ copies/µL) was added, followed by 200 µL of chloroform. The mixture was vortexed and incubated at room temperature for 3 min and then centrifuged at 12,000× *g* for 15 min at 4 °C to separate the phases. The aqueous phase was transferred to a new tube and mixed with 1.5 volumes of 100% ethanol. The solution was applied to a RNeasy MinElute spin column (Qiagen), washed according to the manufacturer’s protocol, and total RNA was finally eluted in 14 µL of RNase-free water. For miRNA analysis, isolated miRNAs were reverse-transcribed into cDNA using the Mir-X miRNA First-Strand Synthesis Kit (Takara Bio, Shiga, Japan) according to the manufacturer’s instructions. The primer sequences used for qRT-PCR are listed in Table 4.

### 4.5. miRNA Array Analysis

Total RNA was extracted from human placental tissues using the TRIzol reagent (Ambion) according to the manufacturer’s instructions. After homogenization, the lysate was transferred to microcentrifuge tubes and centrifuged to remove insoluble debris. The supernatant containing RNA was collected, mixed with chloroform, followed by centrifugation at 12,000× *g* for 15 min at 4 °C. The aqueous phase was transferred to a new tube, and RNA was precipitated with isopropanol and recovered by centrifugation at 12,000× *g* for 10 min at 4 °C. The RNA pellet was washed with 75% ethanol and centrifuged at 7500× *g* for 5 min at 4 °C. The resulting RNA pellet was dissolved in nuclease-free water, and RNA quality and integrity were assessed using an Agilent 2100 Bioanalyzer (Agilent Technologies, Santa Clara, CA, USA). Gene expression profiling was performed using the GeneChip^®^ Prime View^TM^ Human Gene Expression Array (Affymetrix), which contains over 530,000 probes representing approximately 20,000 well-annotated human genes. Each gene is represented by multiple oligonucleotide probe pairs synthesized directly on the array.

### 4.6. cDNA Microarray Analysis

Biotin-labeled cDNA was synthesized from 500 ng of total RNA according to the standard Affymetrix protocol (Expression Analysis Technical Manual, Affymetrix Inc., Santa Clara, CA, USA). After fragmentation, 12 µg of labeled cDNA was hybridized to the GeneChip Human Genome Array for 16 h at 45 °C. Following hybridization, arrays were washed and stained using the Affymetrix Fluidics Station 450 and scanned with Affymetrix GeneChip Scanner 3000 7 G. Raw microarray data were processed using Robust Multi-array Average (RMA) normalization with default Affymetrix analysis settings. The trimmed mean target intensity of each array was scaled to a value of 100 for normalization. The normalized and log-transformed expression values were subsequently analyzed using GeneSpring GX 13.0 software (Agilent Technologies). Differentially expressed genes were selected based on fold-change thresholds, with genes showing ≤0.66-fold downregulation relative to control samples considered significant. Hierarchical clustering analysis was performed using Euclidean distance and average linkage methods in GeneSpring GX 13.0 software (Agilent Technologies, Santa Clara, CA, USA) to identify gene expression patterns across samples.

### 4.7. Quantitative Real-Time PCR Analysis for mRNA and miRNA

For mRNA analysis, cDNA was amplified with *PCYT1B*-specific primers and detected with SYBR Green master mix (Hoffmann-La Roche, Basel, Switzerland) using an Exicycler^TM^ 96 real-time PCR system (Bioneer, Daejeon, Republic of Korea). The qRT-PCR amplification conditions were as follows: initial denaturation at 95 °C for 5 min, followed by 45 cycles of 95 °C for 5 s, and 59 °C for 30 s. All reactions were performed in duplicate. Relative gene expression levels were calculated using the 2^−∆∆Ct^ method after normalization to GAPDH as an internal control.

For miRNA analysis, 2 µL of synthesized cDNA was used as the template for qRT-PCR using miRNA-specific forward primers and SYBR Green master mix (Hoffmann-La Hoffmann-La Roche, Basel, Switzerland). PCR amplification was performed under the following conditions: initial denaturation at 95 °C for 10 min, followed by 40 cycles of 95 °C for 10 s, 95 °C for 5 s and 60 °C for 20 s. A melting curve analysis was performed to verify amplification specificity. All reactions were conducted using the Exicycler^TM^ 96 Real-Time PCR system. Relative miRNA expression levels were calculated using the 2^−ΔΔCt^ method, with U6 small nuclear RNA used as the internal control.

### 4.8. Plasmid Construction for Luciferase Assay

The 3′ untranslated regions (3′UTRs) of target genes containing predicted miRNA binding sites were identified using the miRanda algorithm. Oligonucleotides corresponding to the predicted binding regions were designed for luciferase reporter assays. Fragments of the target gene 3′UTR were amplified by PCR cloning h-Taq DNA polymerase (Solgent Co., Daejeon, Republic of Korea). The PCR reaction mixture contained 10× Taq buffer, 10 mM dNTP mix, and h-Taq polymerase. PCR amplification was performed under the following conditions: initial denaturation at 95 °C for 5 min, followed by 35 cycles of 95 °C for 5 s, 60 °C for 30 s, and 72° C for 1 min, with a final hold at 4 °C. The amplified PCR products were purified using a PCR purification kit (Bioneer, Daejeon, Republic of Korea). The purified fragments were inserted into the multiple cloning site of the pmirGLO vector (Promega, Madison, WI, USA), which had been linearized using restriction enzymes. The ligation mixture containing the linearized pmirGLO vector and PCR product was prepared according to the manufacturer’s protocol and transformed into competent Escherichia coli cells. Transformed cells were plated on ampicillin-containing agar plates and incubated overnight. Colonies were selected and cultured in ampicillin-containing LB growth, and plasmid DNA was extracted using a plasmid miniprep kit (Bioneer). The integrity of the inserted sequences was confirmed by DNA sequencing (Bioneer)

### 4.9. Dual-Luciferase Reporter Assay

To verify the interaction between miRNAs and the 3′ untranslated region (3′UTR) of the target gene, a dual-luciferase reporter assay was performed. HeLa cells were seeded in 24-well plates at a density of 5 × 10^4^ cells/well and cultured until approximately 80% confluence. Cells were then co-transfected with the pmirGLO reporter vector (Promega, Madison, WI, USA) containing the target 3′UTR and miRNA mimics using Lipofectamine 2000 (Thermo Fisher Scientific) according to the manufacturer’s protocol. For each transfection, 150 ng of pmirGLO plasmid DNA was used either alone or in combination with 20 nM miRNA mimic or negative control miRNA. Transfection complexes were prepared in Opti-MEM medium (Gibco, Rockville, MD, USA). After 24 h of transfection, the culture medium was removed, and the cells were washed with Dulbecco’s phosphate-buffered saline (DPBS; Hyclone). Cells were then lysed using 1× Passive Lysis Buffer (PLB) (Promega, Madison, WI, USA). Specifically, 100 µL of PLB per well was added to the 24-well plates, followed by incubation on an orbital shaker at room temperature for 15 min. Cell lysates (20 µL) were transferred to a 96-well white plate (Promega), and 80 µL of Luciferase Assay Reagent II (LAR II) (Promega)) was added to measure Firefly luciferase activity using a luminometer (BioTek Instruments, Winooski, VT, USA). Subsequently, 80 µL of Stop & Glo^®^ reagent (Promega) was added to each well to measure Renilla luciferase (*R Luc*) activity. Relative luciferase activity was calculated as the ratio of Firefly luciferase activity to Renilla luciferase (*R Luc*) activity.

### 4.10. Cell Invasion Assay

To evaluate the invasion ability of trophoblast cells following miRNA mimic or inhibitor treatment, HTR-8/SVneo cells (1 × 10^4^ cells/well) were seeded into 24-well trans-well inserts after transfection with miRNA mimics or inhibitors for 24 h. Invaded trophoblast cells were stained and quantified.

### 4.11. Immunofluorescence Staining

Human placental tissues were sectioned at 8–10 μm thickness and fixed in methanol for 10 min. After fixation, tissue sections were washed with 1× phosphate-buffered saline (PBS) and incubated with blocking solution (Dako, Glostrup, Denmark) for 1 h at room temperature. Sections were then incubated overnight at 4 °C with the following primary antibody diluted in antibody dilution buffer (Dako): Rabbit anti-PCYT1B antibody (NBP2-19734, Novus biologicals, Centennial, CO, USA). After primary antibody incubation, sections were washed with PBS and incubated with fluorescent secondary antibodies for 1 h at room temperature. Finally, tissue sections were mounted using VECTASHIELD mounting medium containing DAPI (Vector Laboratories, Burlingame, CA, USA). Fluorescence images were obtained using a fluorescence microscope (EVOS^TM^, Thermo Fisher Scientific) and representative images were captured.

### 4.12. Statistical Analysis

All experiments were performed in duplicate or triplicate and repeated in at least three independent experiments. Data are presented as mean ± standard error of the meaning (SEM). Statistical significance was determined using Student’s *t*-test. A *p*-value <0.05 was considered statistically significant.

## 5. Conclusions

In conclusion, this study identified PCYT1B and its associated miRNAs as novel regulatory factors related to preterm labor (PTL) and preeclampsia (PE). PCYT1B expression was significantly elevated in placental tissues and maternal circulation, while several miRNAs directly regulated PCYT1B expression and trophoblast invasion capacity. These findings suggest that the PCYT1B-miRNA axis may contribute to placental dysfunction and abnormal placentation in obstetrical diseases. Furthermore, combined assessment of PCYT1B and its associated miRNAs may serve as a promising strategy for biomarker development and future diagnostic applications in PTL and PE.

## Figures and Tables

**Table 1:** Clinical characteristics of PE cohort.

Parameter	Normal Pregnancy	PE
Maternal age (year)	34 ± 2	30.2 ± 1.1
Gestational age (weeks)	38 ± 2.5	37 ± 3.4
Systolic BP (mmHg)	115 ± 3	134 ± 7
Diastolic BP (mmHg)	72 ± 2	91 ± 2
Mean arterial pressure (MAP)	86.3	105.3
Proteinuria	Negative	≥2+

**Table 2:** Clinical characteristics of PTL cohort.

Parameter	Term	PTL	
Delivery status	Term	≤35 weeks	
Inflammation	-	-	+

**Table 3:** The primers used to determine the expression of genes by qRT-PCR in this study.

Gene	Forward Primer	Reverse Primer
*PCYT1B*	5′-AGG CCA TCA GGA GTG CAT CA-3′	5′-CTG GCA GTT GGT TTC ATC AGC-3′
*RhoA*	5′-TGG AAA GCA GGT AGA GTT GG-3′	5′-GAC TTC TGG GGT CCA CTT TT-3′
*Rac1*	5′-TGA TGC AGG CCA TCA AGT GT-3′	5′-AGA ACA CAT CTG TTT GCG GAT AG-3′
ROCK	5′-GAT CTT GTA GCT CCC GCA TCT GT-3′	5′-GAA GAA AGA GAA GCT CGA GA-3′

**Table 4:** The miRNA primers used to determine the expression of genes by qRT-PCR in this study.

Gene	Forward Primer	Accession
hsa-miR-3065-3p	UCA GCA CCA GGA UAU UGU UGG AG	MIMAT0015066
hsa-miR-4660	UGC AGC UCU GGU GGA AAA UGG AG	MIMAT0019728
hsa-miR-6752-5P	UCC CUG CCC CC AUA CU CCC AG	MIMAT0027404
hsa-miR-6842-5P	UUG GCU GGU CUC UGC UCC GCA G	MIMAT0027587
hsa-miR-7110-5p	CUG GCA GGG GGA GAG GUA	MIMAT0028211

## Data Availability

The original contributions presented in this study are included in the article/Supplementary Material. Further inquiries can be directed to the corresponding author.

## Supplementary Materials

The following supporting information can be downloaded at: https://www.mdpi.com/article/10.3390/ijms27094039/s1.
