# Supplementary material for: PCYT1B-Targeting miRNAs as Potential Biomarkers for Placental Diseases
Source: Int J Mol Sci. 2026 Apr 30;27(9):4039. doi: 10.3390/ijms27094039 (PMC13164417; doi:10.3390/ijms27094039)
Supplement: Supplementary file 1 [file ijms-27-04039-s001.zip › ijms-4258689-supplementary.pdf]

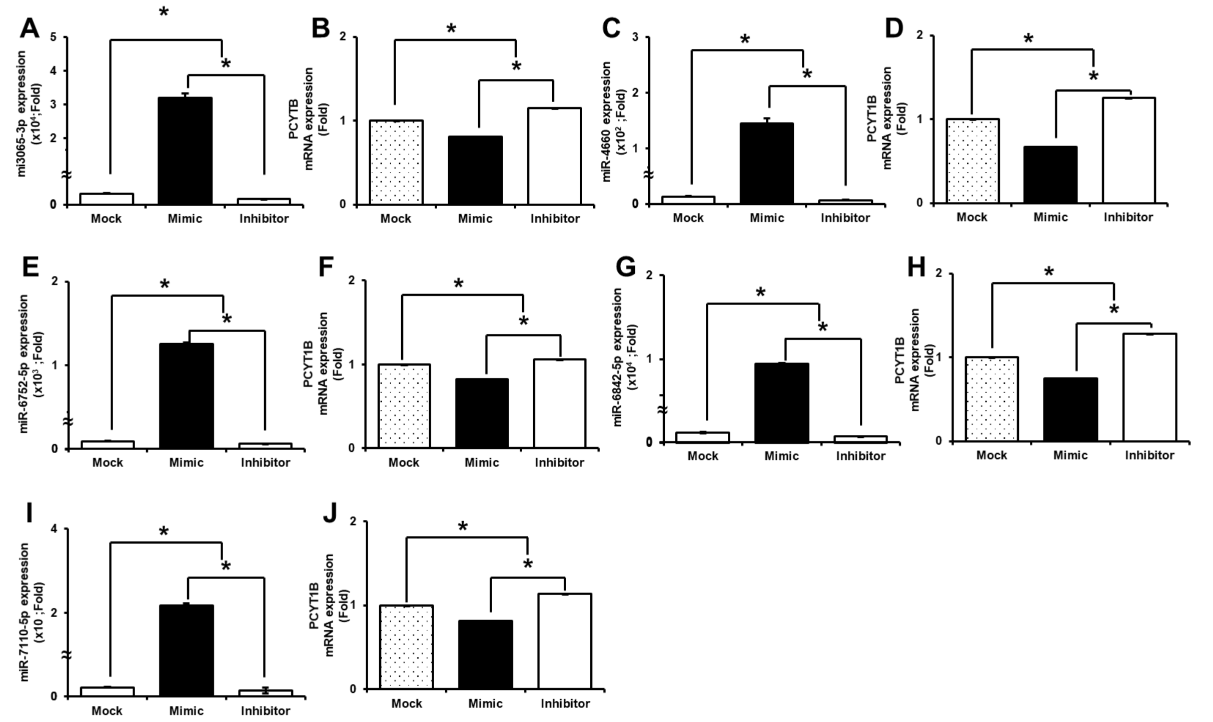

Figure S1. Regulatory effects of selected miRNAs on PCYT1B expression in HTR-8/SVneo trophoblast cells. HTR-8/SVneo cells were transfected with miRNA mimics or inhibitors targeting PCYT1B. Relative expression levels of each miRNA and corresponding PCYT1B mRNA were anlayzed by qRT-PCR. (A, B) miR-3065-3p and PCYT1B, (C, D) miR-4660p and PCYT1B, (E, F) miR-6752-5p and PCYT1B, (G, H) miR-6842-5p and PCYT1B, (I, J) miR-7110-5p and PCYT1B. White bars indicate mimic-treated cells, black bars indicate inhibitor-treated cells, and dotted bars indicate mock controls. Data are presented as mean ± SEM. Statistical significance was determined using Student's t-test. $p < 0.05$ was considered statistically significant.

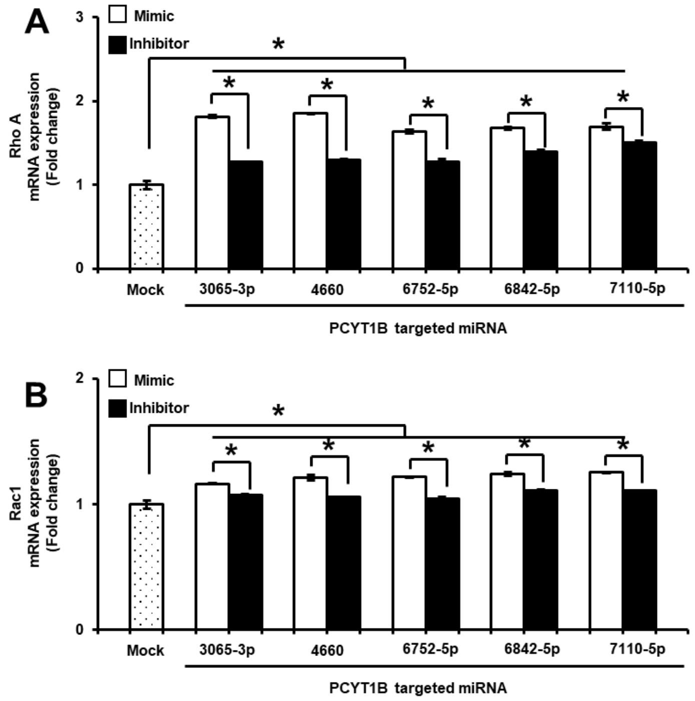

Figure S2. Effect of PCYT1B-targeting miRNAs on migration-related gene expression in trophoblast cells. HTR-8/SVneo trophoblast cells were transfected with miRNA mimics or inhibitors targeting PCYT1B. Relative mRNA expression levels of migration-related genes were analyzed by qRT-PCR: (A) RhoA and (B) Rac1. Data presented as mean ± SEM. Statistical analysis was performed using the Student's t-test. $p < 0.05$ was considered statistically significant.
